# Addition of tumour infiltration depth and extranodal extension improves the prognostic value of the pathological TNM classification for early‐stage oral squamous cell carcinoma

**DOI:** 10.1111/his.13886

**Published:** 2019-07-29

**Authors:** Koos Boeve, Lieuwe J Melchers, Ed Schuuring, Jan L Roodenburg, Gyorgy B Halmos, Boukje A van Dijk, Bert van der Vegt, Max J Witjes

**Affiliations:** ^1^ Department of Oral and Maxillofacial Surgery University of Groningen Groningen The Netherlands; ^2^ Department of Pathology & Medical Biology University of Groningen Groningen The Netherlands; ^3^ Department of Otorhinolaryngology/Head & Neck Surgery University of Groningen Groningen The Netherlands; ^4^ Department of Epidemiology University Medical Centre Groningen, University of Groningen Groningen The Netherlands; ^5^ Department of Research Comprehensive Cancer Organization of The Netherlands (IKNL) Utrecht The Netherlands

**Keywords:** head and neck cancer, lymph nodes, mouth neoplasms, oral cancer, survival, TNM staging

## Abstract

**Aims:**

In the 8th edition of the American Joint Committee on Cancer TNM staging manual, tumour infiltration depth and extranodal extension are added to the pathological classification for oral squamous cell carcinoma. The currently available 8th TNM validation studies lack patients with conservative neck treatment, and changes in the classification especially affect patients with small tumours. The aim of this study was to determine the potential impact of the changes in the 8th edition pTNM classification on the prognosis and treatment strategy for oral squamous cell carcinoma in a well‐defined series of pT1–T2 patients with long‐term follow‐up.

**Methods and results:**

Two hundred and eleven first primary pT1–T2 oral squamous cell carcinoma patients, with surgical resection as primary treatment, were analysed retrospectively. One hundred and seventy‐three patients underwent a neck dissection, and 38 patients had frequent clinical neck assessments. Long‐term follow‐up (median 64 months) and reassessed tumour infiltration depth were available. Classification according to the 8th edition criteria resulted in 36% total upstaging with the T classification and 16% total upstaging with the N classification. T3‐restaged patients (*n* = 30, 14%) had lower 5‐year disease‐specific survival rates than T2‐staged patients (81% versus 67%, *P* = 0.042). Postoperative (chemo)radiotherapy could have been considered in another seven (3%) patients on the basis of the 8th edition criteria.

**Conclusions:**

Addition of tumour infiltration depth and extranodal extension in the 8th TNM classification leads to the identification of oral squamous cell carcinoma patients with a worse prognosis who might benefit from an improved postoperative treatment strategy.

## Introduction

In 2016, the 8th edition of the American Joint Committee on Cancer (AJCC) TNM staging manual was released.^1^ As compared with the 7th edition, tumour infiltration depth and extranodal extension (ENE) were incorporated into the pathological TNM classification for oral squamous cell carcinoma (OSCC).^1^, ^2^ On the basis of the 8th edition criteria, 7th edition pT1 patients with a tumour infiltration depth between 5 and 10 mm are restaged as pT2, and all pT1 and pT2 patients with a tumour infiltration depth of >10 mm are restaged as pT3. Following the pN classification in the 8th edition, cases with a single positive lymph node <30 mm in diameter with ENE are restaged from pN1 to pN2b, and all other ENE‐positive patients are restaged as pN3b.

The incorporation of tumour infiltration depth and ENE in the pathological TNM classification was based on data from both the International Consortium for Outcome Research in Head and Neck Cancer (ICOR) (*n* = 3149) and the National Cancer Data Base (*n* = 7264).^2^, ^3^ The 8th edition has been validated in various independent databases: the pT and pN classifications by Lydiatt *et al*.^2^ (*n* = 1792) and Matos *et al*.^4^ (*n* = 298), and the pN classification by Garcia *et al*.^5^ (*n* = 1137). These studies confirmed a better prediction of survival per stratification with the 8th pTNM classification edition, whereby patients who had been upstaged because of the incorporation of tumour infiltration depth and ENE generally had lower survival rates.

Despite the validation with big data, the clinical impact for small tumours (pT1–T2) is not really clear. As mentioned by Matos *et al*.^3^, ^4^ and the ICOR study, their populations were limited to patients undergoing neck dissections. Patients with a clinically negative neck not treated with selective neck dissections—also known as watchful waiting—were not included. This point is important, because incorporation of infiltration depth in the pT classification could also influence prognosis and, as a result, change the treatment strategy for these early‐stage patients. Therefore, our aim was to study the clinical impact of the 8th edition pTNM classification on the survival of 7th edition pT1–T2 patients treated with surgical resection of the tumour combined with neck dissection or a watchful waiting strategy. We selected pathologically staged T1–T2 OSCC patients from our large and homogeneous database with extensive clinicopathological and long‐term follow‐up data.^6^, ^7^

## Materials and methods

### Patients

This cohort with reassessed tumour infiltration depth has been previously described.^6^, ^7^ Briefly, 246 consecutive patients with pT1–T2 OSCC according to the 7th edition, diagnosed between 1997 and 2008 with a first primary tumour and treated with surgical resection of the tumour at the University Medical Centre Groningen, were selected from our database. Thirty‐five patients were excluded because of multiple head and neck tumours (*n* = 3), irretrievable haematoxylin and eosin (H&E) slides (*n* = 13), or unreliable assessment of infiltration depth because of missing epithelial surfaces and tangential tissue cutting (*n* = 19), resulting in 211 patients being available for tumour infiltration depth reassessment. Thirty‐eight patients (18%) with a pT1 tumour did not undergo a neck dissection, but were followed closely (watchful waiting). This strategy was common in the era before the awareness that an infiltration depth of 4 mm implied a high chance of tumour spread to lymph nodes.^6^ The 38 patients with watchful waiting had a median tumour infiltration depth of 3.2 mm [interquartile range (IQR) 2.1–5.6 mm]. In total, 211 patients were used for analysis, and 173 of these were treated with neck dissection. The clinical and histopathological characteristics of the study group are shown in Table 1. In total, 72 patients received postoperative radiotherapy, but none of the watchful waiting patients were postoperatively irradiated. The median follow‐up time was 64 months (range 0–193 months). Thirteen patients (6%) were diagnosed with local recurrence and 26 (12%) with regional recurrence. Of the 38 watchful waiting patients, two patients were diagnosed with a local recurrence and seven patients with regional recurrences during their follow‐up. Sixty‐eight patients (32%) died in the first 5 years after treatment, 57% because of the OSCC. OSCC‐related death (median 63 years; IQR 54–70 years) occurred at a significantly younger age than OSCC‐unrelated death (median 71 years; IQR 62–79 years) (*P* = 0.010).

**Table 1:** Population characteristics

Variables	Value
Total patients, *n* (%)	211 (100)
Gender, *n* (%)	
Male	118 (56)
Female	93 (44)
Age at diagnosis (years)	
Mean (SD)	62 (13)
Range	25–94
Site, *n* (%)	
Tongue	108 (51)
Gum	14 (7)
Floor of mouth	64 (30)
Cheek mucosa	7 (3)
Retromolar area	12 (6)
Other	6 (3)
cT status (7th edition), *n* (%)	
1–2	189 (90)
3–4	22 (10)
cN status (7th edition), *n* (%)	
cN+	50 (24)
cN0	161 (76)
Histopathological characteristics	
Tumour thickness (mm)	
Median (IQR)	6.0 (3.3–9.0)
Range	0.1–20.0
Perineural invasion, *n* (%)	35 (17)
Lymphovascular invasion, *n* (%)	19 (9)
Involved margins (<1 mm)	32 (15)
PO(C)RT, *n* (%)	72 (34)
Follow‐up (months)	
Median (IQR)	64 (30–99)
Range	0–193
Recurrences, *n* (%)	
Locoregional recurrence	13 (6)
Regional recurrence	26 (12)
Distant metastasis	6 (3)
Death, *n* (%)	
Due to disease	36 (17)
Overall	68 (32)

IQR, interquartile range; PO(C)RT, postoperative chemotherapy or radiation therapy; SD, standard deviation.

### Data Collection

Clinical and pathological data were collected retrospectively from the patient files. Tumour H&E‐stained slides were revised by one dedicated head and neck pathologist, and tumour infiltration depth was reassessed by the use of digital microscopy and computerised measurements (research assistant 6; RVC, Soest, The Netherlands). Tumour infiltration depth was measured from the mucosal surface or from the reconstructed mucosal surface in cases of ulcerated or exophytic tumours^7^; this differs from the AJCC manual in using the mucosal surface instead of the mucosal basement membrane.^2^ ENE was defined as an extension of tumour cells beyond the nodal capsule, and forms part of the standard pathology report in our centre. Cases with no convincing extension beyond the nodal capsule (i.e. no stromal reaction) were scored as negative. We revised the pathological tumour and pathological nodal classification according to the 8th edition. Five‐year disease‐specific survival (DSS) was defined as the time from first treatment until disease‐specific death or the last follow‐up, with a maximum of 5 years. Three‐year disease‐free survival (DFS) was defined as the time until local, regional or distant recurrence or the last follow‐up within 3 years after the start of the initial treatment. Death was censored and did not count as a DFS event.

### Ethical Justification

As this study used retrospectively evaluated data from patients treated according to the Dutch national guidelines for oral cavity cancer, approval from the hospital research ethics board was not necessary according to the Dutch ethical regulations.^8^

### Statistics

Categorical data are presented as number and percentage, normally distributed data are presented as mean with standard deviation (SD), and skewed data are presented as median with IQR. Fisher’s exact or chi‐squared tests were used to test the associations between categorical data. The log‐rank test was used to analyse differences between the Kaplan–Meier curves. DSS is reported as a percentage of survival after 5 years, and DFS is reported as a percentage of survival after 3 years. stata statistical software (Release 15.1) was used to determine the 95% confidence intervals of the DSS and DFS survival percentages (Stata Corp., College Station, TX, USA). All other statistical analyses were performed with ibm spss statistics 23 (SPSS, Chicago, IL, USA). *P*‐values of <0.05 were considered to be significant for all of the statistical analyses.

## Results

### Tumour Staging

**Figure 1:**
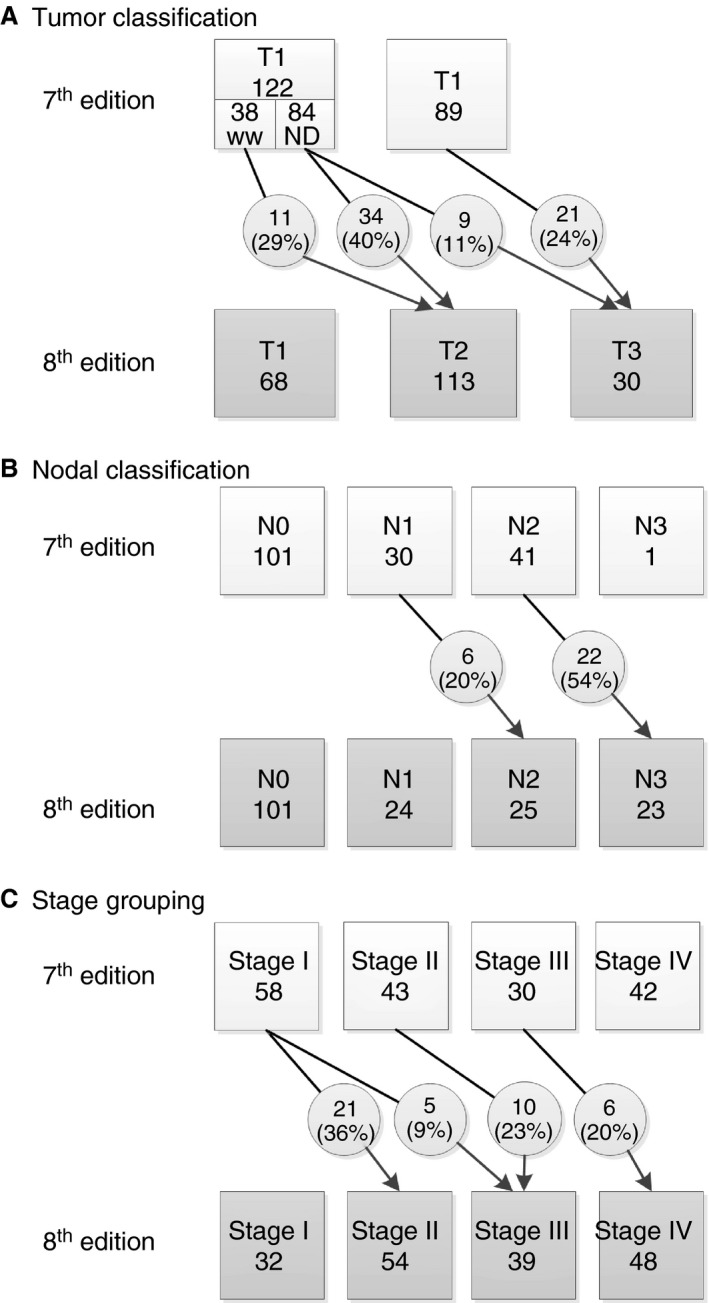
Differences in tumour, nodal and stage grouping between the 7th and 8th editions of the American Joint Committee on Cancer TNM classification. N, nodal; ND, neck dissection; T, tumour; WW, watchful waiting.

**Figure 2:**
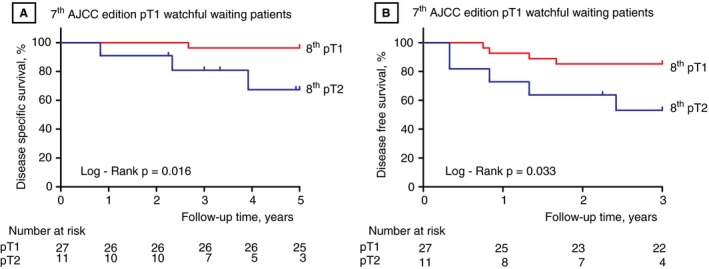
Differences in disease‐specific survival (**A**) and disease‐free survival (**B**) for 7th pT1‐classified patients with a watchful waiting strategy of the neck which were staged using the 8th edition pT criteria

**Figure 3:**
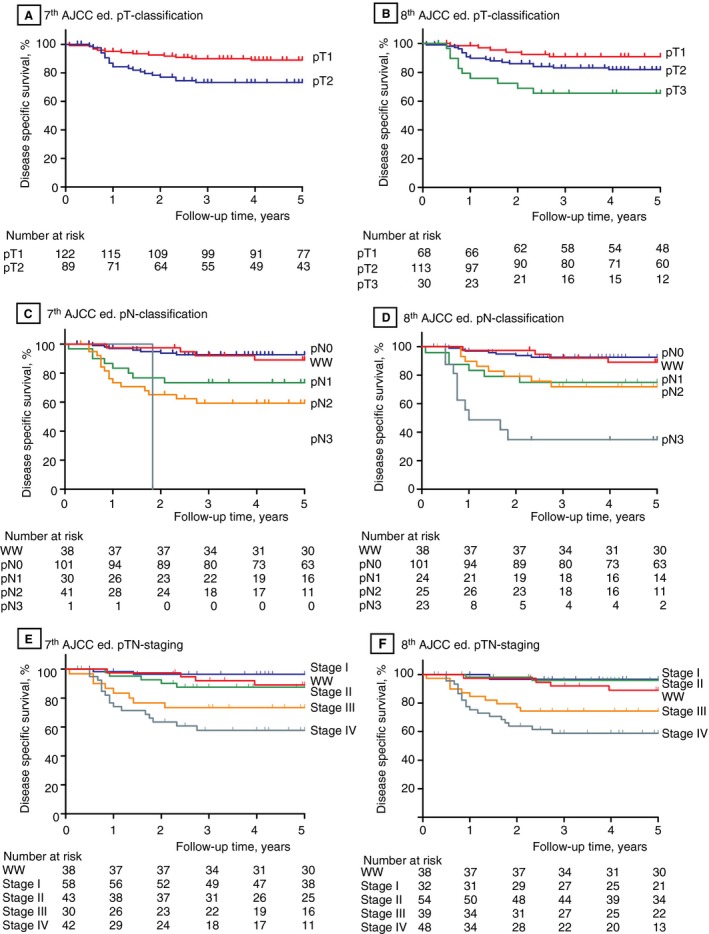
Disease‐specific survival (DSS) Kaplan–Meier curves for the 7th edition (**A,C,E**) and the 8th edition (**B,D,F**) American Joint Committee on Cancer TNM classifications. Five‐year survival rates with their 95% confidence intervals for each disease‐specific survival curve and log‐rank test are given in Table 2. N, nodal; T, tumour; WW, watchful waiting.

In total, 211 patients with a median tumour infiltration depth of 6.0 mm (IQR 3.3–9.9 mm) were used for the pT classification analysis. Tumour restaging according to the 8th edition resulted in upstaging of 75 (36%) of the 211 patients: 12 (6%) patients on the basis of both tumour infiltration depth and ENE, and 63 (30%) patients on the basis of tumour infiltration depth only. Figure 1A shows the differences between the 7th and 8th pT editions. Fifty‐four pT1 patients (44%) and 21 pT2 patients (24%) were restaged according to the 8th edition criteria. Patients with tongue tumours were significantly more often restaged to pT2 (31%) or pT3 (19%) than patients with tumours in the other anatomical locations, for whom restaging occurred in 12% (pT2) and 9% (pT3) (*P* < 0.001). Of the 38 watchful waiting patients, 11 (29%) were restaged to pT2. These patients had significantly shorter DSS (*P* = 0.016) and DFS (*P* = 0.033) than the other 27 patients (Figure 2A, B). Within the watchful waiting group, three (11%) of the 27 non‐restaged patients and four (36%) of the 11 restaged patients were diagnosed with regional recurrences during follow‐up (not significant). Sixteen of the 45 patients (35%) restaged from pT1 to pT2 had undergone postoperative radiotherapy after surgical resection of the tumour. Twenty‐three of the 30 patients restaged as pT3 had been postoperatively irradiated. The 8th edition pT classification showed good stratification, with significantly shorter DSS for the pT1–T2 patients upstaged to pT3 than for non‐restaged pT2 patients (81% versus 66%, *P* = 0.048; Figure 3B; Table 2).

**Table 2:** Disease‐specific survival rates, 95% confidence intervals (CIs) and log‐rank test results of all stages

Edition	Category	Survival		Log‐rank test	
		5‐year (%)	95% CI	Compared groups	*P*‐value
7th pT class	T1	89	82–93	T1 versus T2	0.002
	T2	72	61–81		
8th pT class	T1	91	81–96	T1 versus T2	0.077
	T2	81	72–87	T2 versus T3	0.048
	T3	66	45–80	T1 versus T3	0.001
7th pN class	WW	89	73–86	WW versus N0	0.734
	N0	92	84–96	N0 versus N1	0.005
	N1	73	54–86	N1 versus N2	0.264
	N2	59	42–73	N2 versus N3	0.402
	N3	0	NA		
8th pN class	WW	89	73–96	WW versus N0	0.734
	N0	92	84–96	N0 versus N1	0.016
	N1	75	53–88	N1 versus N2	0.793
	N2	69	46–84	N2 versus N3	0.072
	N3	48	26–67		
7th SG class	WW	89	73–96	WW versus stage I	0.195
	Stage I	96	86–99	Stage I versus stage II	0.056
	Stage II	85	70–93	Stage II versus stage III	0.184
	Stage III	73	54–86	Stage III versus stage IV	0.220
	Stage IV	58	40–72		
8th SG class	WW	89	73–96	WW versus stage I	0.270
	Stage I	97	78–99	Stage I versus stage II	0.594
	Stage II	94	83–98	Stage II versus stage III	0.007
	Stage III	74	58–85	Stage III versus stage IV	0.167
	Stage IV	59	43–72		

N, nodal; SG, stage grouping; T, tumour; WW, watchful waiting.

### Nodal Staging

Of the 173 neck dissection patients, 72 (42%) were diagnosed with nodal metastasis. Twenty‐eight (16%) of these 173 patients were restaged with the 8th edition criteria because of ENEs (Figure 1B). No significant differences were seen in N status restaging between anatomical locations. Twenty‐six of the 28 restaged patients had been treated postoperatively with radiotherapy, which was combined with chemotherapy in one patient. The 8th edition pN classification showed good stratification, with an 89% 5‐year survival rate for the watchful waiting patients, and pN3‐staged patients having the shortest survival rates, although the difference in DSS between pN2‐staged and pN3‐staged patients was not significant (69% versus 48%, *P* = 0.072; Figure 3D; Table 2).

### Stage Grouping

In total, 42 (20%) patients were restaged with the 8th edition criteria. Differences in stage grouping per category between the 7th and 8th editions are shown in Figure 1C. Restaging with the 8th edition resulted in a smaller difference in DSS between stage I and stage II: 11% versus 3% difference in the 5‐year DSS between the 7th and 8th stage I and stage II editions, respectively. After restaging with the 8th edition criteria, the difference in DSS was larger between stage II and stage III, being 12% with the 7th edition (85% and 73%, not significant) versus 20% with the 8th edition (94% and 74%, *P* = 0.007) (Figure 3F; Table 2).

## Discussion

The aim of this study was to determine the clinical impact of the addition of tumour infiltration depth and ENE in the 8th edition of the pathological TNM classification on survival and potential alterations in treatment strategy for pT1–T2 OSCC patients who had been treated on the basis of the 7th edition. In this study, 36% and 16% of all of the patients were restaged with the 8th edition criteria according to the pT and pN classifications, respectively. Patients restaged as pT3 showed significantly shorter DSS than 8th edition pT1–T2‐staged patients. Another seven (3%) patients who were restaged as pT3 could possibly have benefited from postoperative radiotherapy.

This study used a well‐defined 7th edition pT1–T2 cohort with extensive clinical data to add to the current evidence validating the 8th edition TNM classification.^2^, ^4^, ^5^ Patients with a watchful waiting strategy of the neck were also included, which was not the case in the large ICOR study and the validation study by Matos *et al*.^3^, ^4^ Recently, two other studies investigated the differences between the 7th edition and 8th edition TNM staging by using early‐stage OSCC patients.^9^, ^10^ These studies differed from the current study by using sentinel lymph node biopsy (SLNB)‐staged patients or by analysing only the pT categories and not the pN categories. This study confirms the previously mentioned validation study findings regarding the shorter survival rate of patients restaged as pT3 and pN3 with the 8th edition criteria.^2^, ^4^, ^5^ However, the number of restaged patients differs between studies. In this study, 44% of the 7th edition pT1 patients were restaged, versus 44% and 61% in other studies,^3^, ^4^ and 24% of the pT2 patients were restaged, versus 62% and 47% in other studies.^3^, ^4^ Remarkably, one of the other studies did not restage any of the 7th edition pT1 patients to pT3.^3^ Differences in restaging rates might be explained by differences in clinical care between the countries. In The Netherlands, people visit their general dental practitioner once a year or more, whereas one of the validation studies stated in the discussion that the restaging rates could have been limited by a high rate of advanced disease, which is a reality in emerging countries.^4^

Restaging to a higher classification level with the 8th edition criteria is only possible for 7th edition pT1–T2 patients. Consequently, the 8th edition is clinically most relevant for these patients. This is why we used a cohort of 7th edition pT1–T2 patients to obtain an unadulterated view of the differences in prognosis. The inclusion of only pT1–T2 patients resulted in a relatively small number of 8th edition pT3 patients as compared with other studies. Also, the ENE rate in this study is lower than in the other 8th edition TNM validation studies: 39% versus 51% and 53%, respectively.^4^, ^5^ The inclusion of only pT1–T2 patients could explain the lower ENE rate than in studies that also included more advanced disease.

We previously stated that a tumour infiltration depth of 4 mm could serve as an optimal cut‐off between elective and therapeutic neck dissections, on the basis of results obtained with the same cohort.^6^ Therefore, it is not surprising that the 8th edition pT2 patients (tumour infiltration depth of 5–10 mm) showed shorter survival in this study. Furthermore, another study suggested using a 4‐mm tumour infiltration depth as a cut‐off for pT3 tumours instead of the 8th edition AJCC pT cut‐offs.^9^ Twelve patients in this cohort had watchful waiting of the neck and an infiltration depth of >4 mm, because they were treated before the introduction of the 4‐mm cut‐off in our centre. Exclusion of these 12 patients resulted in 100% 5‐year survival for the remaining watchful waiting patients, and similar survival stratifications for the 7th and 8th pT and pN categories (Data S1 and S2).

The benefit of this cohort was the availability of long‐term follow‐up, because no adjustments were made for OSCC in the 7th pTNM classification edition when it was released in 2009, as compared with the 6th edition.^11^

Additions to the pTNM classification are useful if they can be measured robustly and have a clinical impact. The national guidelines in The Netherlands support postoperative radiotherapy of T3–T4 tumours, even those with clear margins.^12^ If the patients in this cohort had been staged with the 8th edition and treated accordingly, another 3% of the patients would have received postoperative radiotherapy. Although the patients who were restaged according to the 8th edition pT classification criteria showed lower DSS, prospective studies are needed to confirm that radiotherapy is beneficial for these patients. Besides the adjuvant therapy, SLNB is currently used as staging technique for cT1–2N0 patients in our centre.^13^ This study shows that the 30 (15%) patients who were restaged as T3 would not have had an indication for an SLNB according to the 8th edition criteria. Den Toom *et al*.^10^ stated that 8th edition pT3 patients with tumours ≤40 mm in diameter probably benefit from staging of the neck with the SLNB procedure. However, further data are needed to verify whether the SLNB is still a reliable neck‐staging technique for patients restaged from 7th edition pT1–T2 to 8th edition pT3. In our centre, pN3 patients are treated postoperatively with concomitant chemoradiotherapy according to the current guidelines.^12^ Despite the better prognostic value of the 8th edition pN classification, pN staging with the 8th edition would not alter postoperative treatment strategies in our centre.

The growth of OSCCs can occur in an exophytic, an ulcerative or a superficial manner.^2^, ^6^ These differences in surface growth have resulted in various methods of assessment of tumour infiltration depth and thickness in the past.^2^ To prevent underestimation (ulcerative growth) or overestimation (exophytic growth) of the prognosis, for the 8th pT classification tumour infiltration needs to be measured vertically from the reconstructed mucosa by use of the adjacent mucosal basement membrane of the normal epithelium.^2^ In this study, the mucosal surface was used instead of the basement membrane. Healthy epithelial thicknesses are approximately 216 µm (SD 59 µm) for the tongue and 99 µm (SD 22 µm) for the mucosa of the anterior floor of the mouth.^14^ Because of these small differences between healthy mucosal surfaces and basement membranes, it is improbable that tumour infiltration depth assessment by use of the basement membrane would have a large impact on our data. This was confirmed by an earlier study reporting an extremely high correlation between both methods (3.7% pT category difference).^4^ Another study reported a 5.7% difference in pT category when it compared both methods without correcting for exophytic growth.^15^ In cases of metastasis in lymph nodes, all cases with extension of the metastasis through the fibrous capsule into the surrounding tissue should be scored as ENE‐positive.^2^ To study the effect of ENE size in the future, Lydiatt *et al*.^2^ advocate to divide ENE‐positive lymph nodes in lymph nodes with minor ENE (<2 mm) and major ENE (>2 mm and metastasis without recognisable lymph node).

This study demonstrates, in a well‐defined retrospective cohort of 211 pT1–T2 (7th edition) OSCC patients, that the addition of tumour infiltration depth and ENE, as used in the 8th edition of the AJCC pathological TNM classification, identifies a group of restaged patients with a worse prognosis.

## Conflict of Interests

The authors have no conflicts of interest to declare

## Author contributions

Concept, design, analysis and interpretation of data: K. Boeve, B. A. van Dijk, E. Schuuring, J. L. Roodenburg, B. van der Vegt, and M. J. Witjes. Acquisition of data: K. Boeve, L. J. Melchers, G. B. Halmos, J. L. Roodenburg, B. van der Vegt, and M. J. Witjes. Critical revision of the manuscript: all authors. Final approval of the version to be published: K. Boeve, E. Schuuring, Jan L Roodenburg, B. van der Vegt, and M. J. Witjes.

## Supporting information


**Data S1** **.** Disease‐specific survival rates, 95% confidence intervals and log‐rank test results of all stages after exclusion of watchful waiting patients with a tumour infiltration depth of >4 mm.

**Data S2** **.** Disease‐specific survival Kaplan‐Meier curves for the 7th edition (**A**,**C**,**E**) and the 8th edition (**B**,**D**,**F**) TNM classifications after exclusion of watchful waiting patients with a tumourinfiltration depth of >4 mm.
