# Supplementary material for: Addition of tumour infiltration depth and extranodal extension improves the prognostic value of the pathological TNM classification for early‐stage oral squamous cell carcinoma
Source: Histopathology. 2019 Jul 29;75(3):329–37. doi: 10.1111/his.13886 (PMC6851684; doi:10.1111/his.13886)
Supplement: Supplementary file 1 — Data S1 . Disease‐specific survival rates, 95% confidence intervals and log‐rank test results of all stages after exclusion of watchful waiting patients with a tumour infiltration depth of >4 mm. [file HIS-75-329-s001.docx]

| **Edition** | **Category** | **Disease Specific Survival** | | **Log-Rank test** | | |
| --- | --- | --- | --- | --- | --- | --- |
|  |  | **5 yr %** | **95% CI** | **Compared stages** | | **p-value** |
| 7^th^ pT | T1 | 92 | 85 to 96 | T1 vs T2 |  | **0.001** |
|  | T2 | 72 | 61 to 81 |  |  |  |
|  |  |  |  |  |  |  |
| 8^th^ pT | T1 | 92 | 83 to 97 | T1 vs T2 |  | 0.066 |
|  | T2 | 82 | 73 to 89 | T2 vs T3 |  | **0.042** |
|  | T3 | 66 | 45 to 80 | T1 vs T3 |  | **0.001** |
| 7^th^ pN | WW | 100 | NA | WW vs N0 |  | 0.135 |
|  | N0 | 92 | 84 to 96 | N0 vs N1 |  | **0.005** |
|  | N1 | 73 | 54 to 86 | N1 vs N2 |  | 0.264 |
|  | N2 | 59 | 42 to 73 | N2 vs N3 |  | 0.402 |
|  | N3 | 0 | NA | NA |  |  |
|  |  |  |  |  |  |  |
| 8^th^ pN | WW | 100 | NA | WW vs N0 |  | 0.135 |
|  | N0 | 92 | 84 to 96 | N0 vs N1 |  | **0.016** |
|  | N1 | 75 | 53 to 88 | N1 vs N2 |  | 0.793 |
|  | N2 | 69 | 46 to 84 | N2 vs N3 |  | 0.072 |
|  | N3 | 48 | 26 to 67 |  |  |  |
| 7^th^ SG | WW | 100 | NA | WW vs Stage I | | 0.333 |
|  | Stage I | 96 | 86 to 99 | Stage I vs Stage II | | **0.056** |
|  | Stage II | 85 | 70 to 93 | Stage II vs Stage III | | 0.184 |
|  | Stage III | 73 | 54 to 86 | Stage III vs Stage IV | | 0.220 |
|  | Stage IV | 58 | 40 to 72 |  |  |  |
|  |  |  |  |  |  |  |
| 8^th^ SG | WW | 100 | NA | WW vs Stage I | | 0.352 |
|  | Stage I | 97 | 79 to 100 | Stage I vs Stage II | | 0.594 |
|  | Stage II | 94 | 83 to 98 | Stage II vs Stage III | | **0.007** |
|  | Stage III | 74 | 58 to 85 | Stage III vs Stage IV | | 0.167 |
|  | Stage IV | 59 | 43 to 72 |  |  |  |

**Supplementary data 1. Disease specific survival rates , 95% confidence intervals and log-rank test of all stages after excluding watchful waiting patients with a tumor infiltration depth > 4mm**

Abbreviations: CI, confidence interval; N, nodal; T, tumor; NA, not applicable; SG, stage grouping; WW, watchful waiting.
