# Supplementary material for: Addition of tumour infiltration depth and extranodal extension improves the prognostic value of the pathological TNM classification for early‐stage oral squamous cell carcinoma
Source: Histopathology. 2019 Jul 29;75(3):329–37. doi: 10.1111/his.13886 (PMC6851684; doi:10.1111/his.13886)
Supplement: Supplementary file 2 — Data S2 . Disease‐specific survival Kaplan‐Meier curves for the 7th edition (A,C,E) and the 8th edition (B,D,F) TNM classifications after exclusion of watchful waiting patients with a tumourinfiltration depth of >4 mm. [file HIS-75-329-s002.docx]

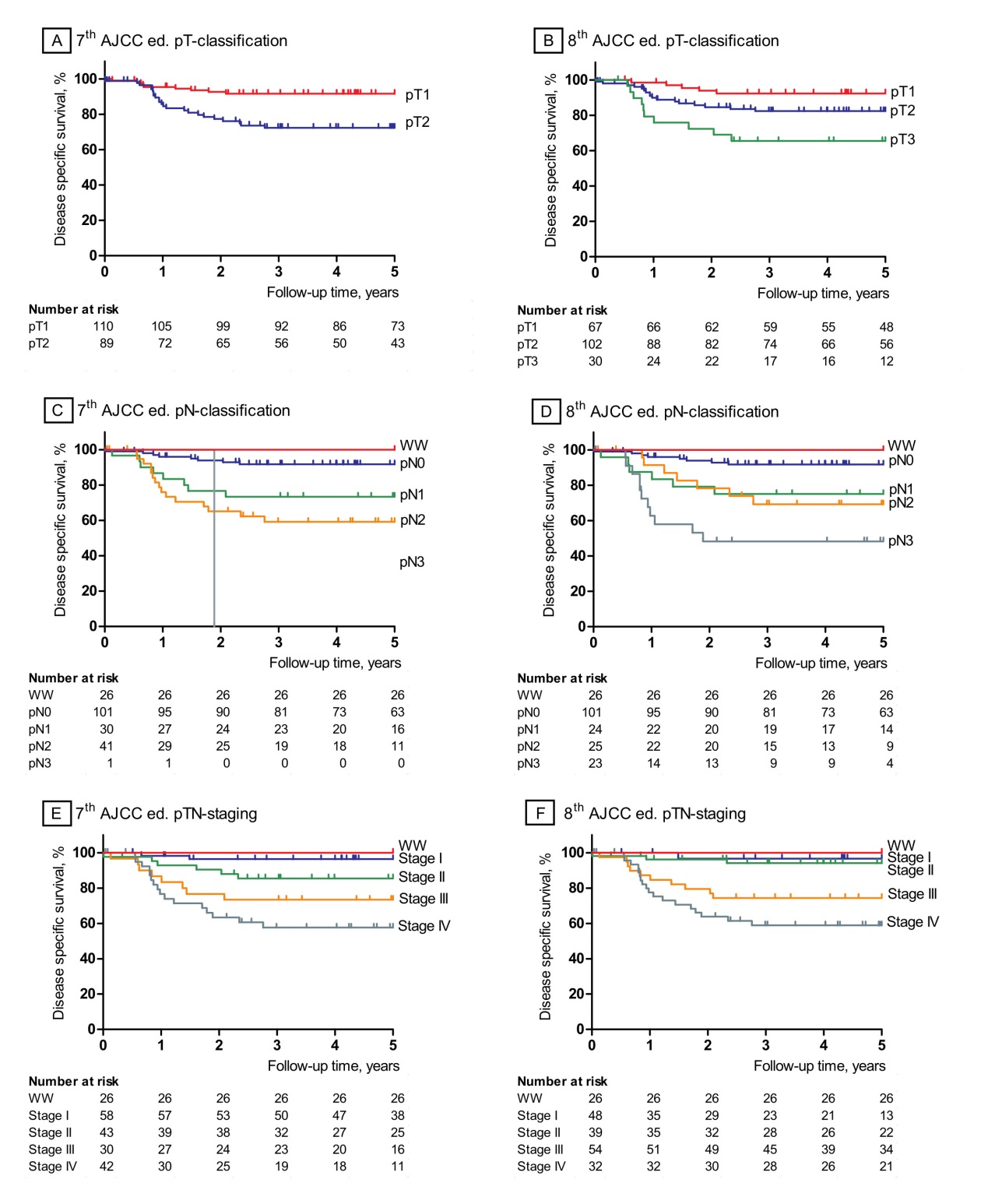
**Supplementary data 2. Disease specific survival Kaplan – Meier curves for the 7^th^ edition (A+C+E) and 8^th^ edition (B+D+F) TNM classifications after excluding watchful waiting patients with a tumor infiltration depth > 4mm**

**Five-year survival rates with their 95% confidence intervals for each disease specific survival curve and log-rank test are given in Table 2.** Abbreviations: AJCC, American Joint Committee on Cancer; ed., edition; DSS, disease specific survival; N, nodal; T, tumor; WW, watchful waiting
